# 663. Long COVID Phenotype Risk Score Reveals Protective Effect of Physical Activity

**DOI:** 10.1093/ofid/ofaf695.218

**Published:** 2026-01-11

**Authors:** Bennett Waxse, Evelynne S Fulda, Josh Denny

**Affiliations:** NIAID, NHGRI, Bethesda, MD; NHGRI, University of Oxford, Bethesda, Maryland; NHGRI, All of Us, Bethesda, Maryland

## Abstract

**Background:**

Long COVID is a heterogeneous and debilitating disease that affects 10-35% of adults after SARS-CoV-2 infection, but little is known about how activity level before and after COVID-19 affects long COVID risk. Using the NIH’s *All of Us* Research Program, we constructed a phenotype risk score (PheRS) predicting long COVID and examined associations between daily step counts and long COVID risk.

**Figure:**
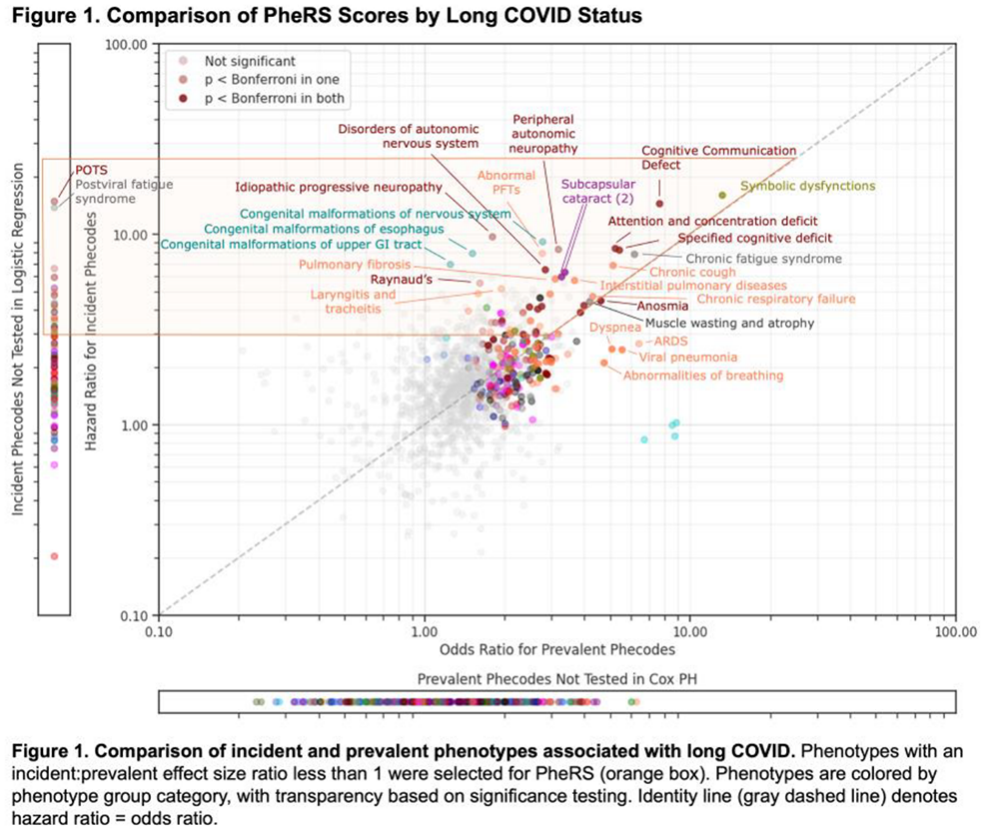

**Figure:**
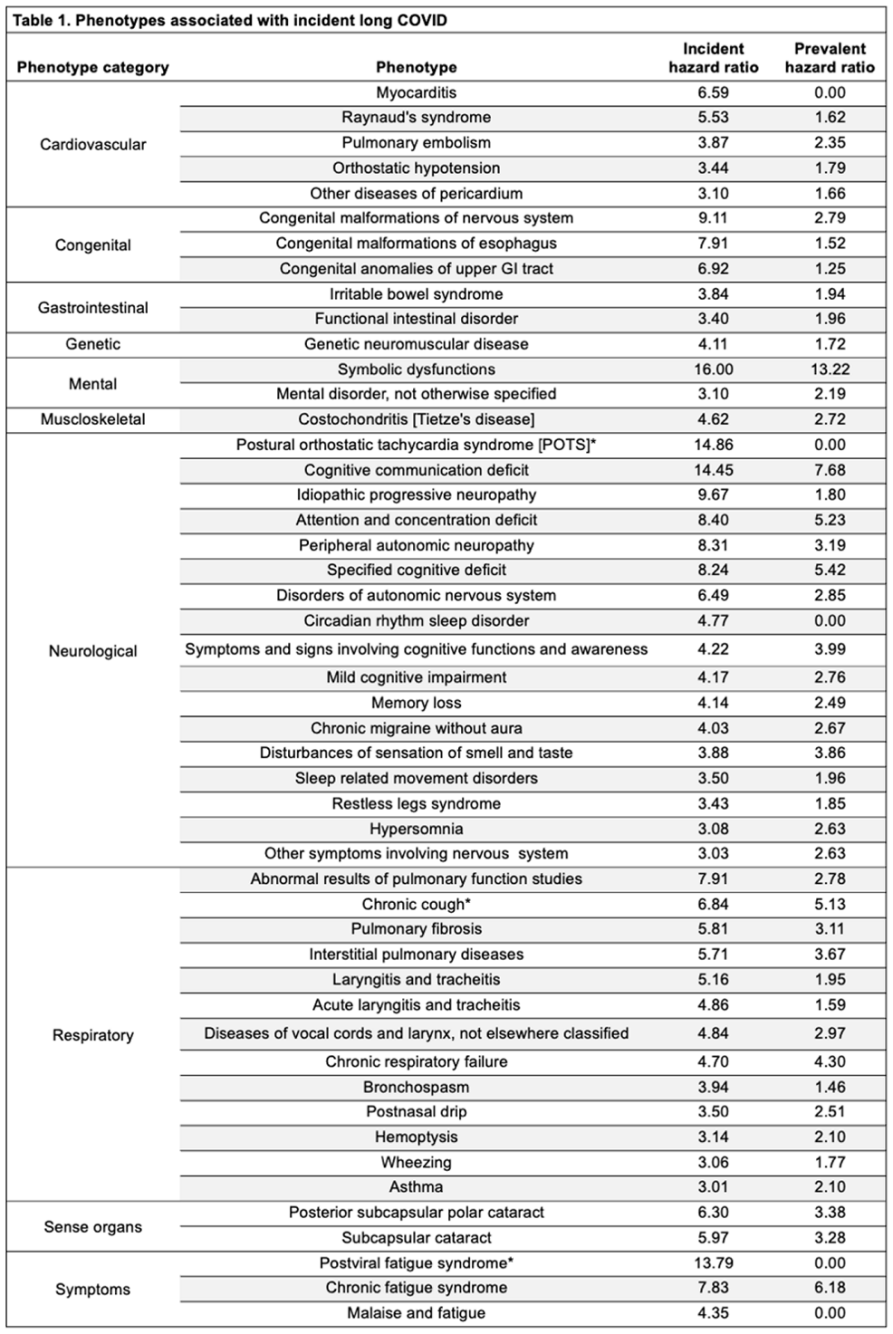

**Methods:**

We identified participants with SARS-CoV-2 infection using EHR data. Using phenome-wide association studies (PheWASs), we compared those with and without long COVID billing codes (U09) 30-365 days post-infection. We constructed a long COVID phenotype risk score (PheRS) from phenotypes with higher effect sizes in the incident versus prevalent PheWAS, comparing it to PheRSs constructed from established long COVID phenotype sets. We assessed relationships between mean daily step counts and long COVID PheRS through regression analyses, adjusting for demographics and infection severity.

**Figure:**
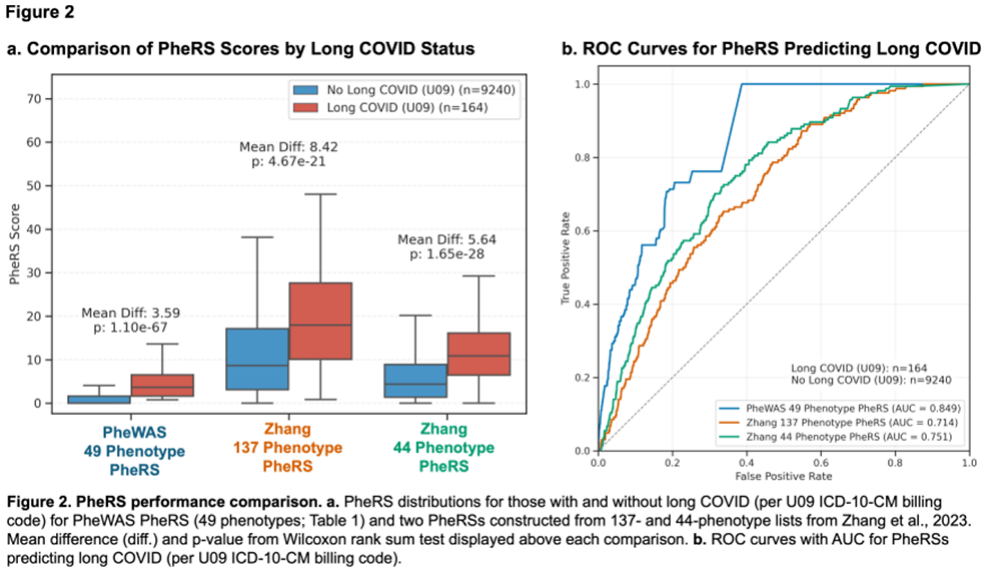

**Figure:**
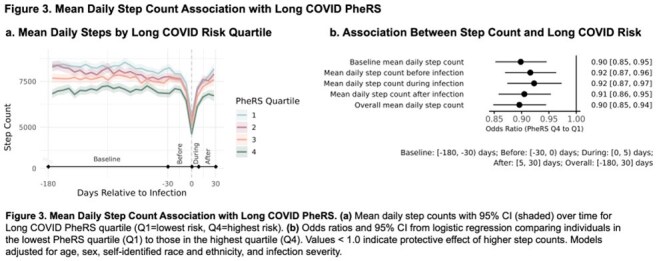

**Results:**

PheWAS comparison revealed distinct phenotype patterns for acute and long COVID-19 (Figure 1). Long COVID phenotypes (i.e. incident:prevalent effect size ratio less than one; orange box, Figure 1) were suggestive of long COVID (Table 1). Our long COVID PheRS outperformed PheRSs constructed from the long COVID phenotypes identified by Zhang et al. (AUC: 0.85 vs 0.75; Figure 2). Higher long COVID risk (higher PheRS quartiles) demonstrated lower mean daily step counts for all time periods (Figure 3a). In adjusted models, higher step counts at all time points were independently associated with lower odds of long COVID risk (OR: 0.90-0.92, all *p*< 0.05; Figure 3b), while changes in step count from baseline were generally not predictive (not shown).

**Conclusion:**

This is the first use of incident PheWAS to create a long COVID PheRS, which outperforms approaches to identify long COVID in other previous EHR algorithms. Findings suggest that maintaining high step counts across all periods may be more important for reducing long COVID risk than higher activity during any single period. This work demonstrates incident PheWAS’s utility for PheRS creation, offers a simple and effective long COVID identification tool for EHR-based research, identifies low baseline activity as an independent risk factor for long COVID.

**Disclosures:**

All Authors: No reported disclosures

